# Intraparenchymal fiberoptic intracranial pressure monitoring and decompressive craniectomy in meningioma case with critical intracranial pressure: A case report during COVID-19 pandemic^☆^

**DOI:** 10.1016/j.ijscr.2022.107364

**Published:** 2022-06-30

**Authors:** Tedy Apriawan, Rizki Meizikri, Endra Wibisono Harmawan, Heru Kustono

**Affiliations:** Department of Neurosurgery, Faculty of Medicine Universitas Airlangga – Dr. Soetomo Academic General Hospital, Surabaya, Indonesia

**Keywords:** DC, Decompressive Craniectomy, ICP, Intracranial Pressure, ICU, Intensive Care Unit, COVID-19, Corona Virus Disease 2019, GCS, Glasgow Coma Scale, CNS, Central Nervous System, TBI, Traumatic Brain Injury, ICP monitoring, Meningioma, Decompressive Craniectomy, Case report

## Abstract

**Introduction:**

Meningioma is a slow-growing tumor that can cause neurological emergency due to intracranial hypertension. The definitive therapy is indeed emergency resection, but it is not always possible in several countries due to limited capacity and/or capability of the emergency operating room. The use of intraparenchymal fiberoptic intracranial pressure (ICP) monitoring and decompressive craniectomy (DC) in cases of brain tumors might be possible, but it is uncommon. We report a meningioma patient in whom immediate meningioma resection was considered too risky due to intensive care unit (ICU) shortage during COVID-19 pandemic and, therefore, underwent these procedures as life-saving measures.

**Case presentation:**

A 24-year-old man was brought to the emergency room with a chief complaint of seizure. Physical examination was notable for decreased consciousness (Glasgow Coma Scale (GCS) 11) and a dilated left pupil with intact light reflex. A contrasted Brain CT Scan revealed extra-axial mass on the left sphenoid with extensive tentacle edema, which pushed the midline structures 2 cm toward the contralateral side.

**Discussion:**

The patient was diagnosed with Left Sphenoid Meningioma. We decided to perform intraparenchymal fiberoptic ICP monitor insertion and DC considering the situation, device availability, safety, and efficacy. The patient slowly regained consciousness in the recovery room after the procedure. The best-observed GCS was 12. Two weeks afterward, the patient came back to our outpatient clinic neurologically intact. The patient was then planned for elective tumor resection.

**Conclusion:**

ICP monitoring and DC are not commonly performed on brain tumor cases. However, in suboptimal situations, these procedures might save lives. The present case showed that ICP monitor and DC were helpful in times of ICU shortage.

## Highlights

• Meningioma is a slow-growing tumor that can cause neurological emergency due to intracranial hypertension.
• The definitive therapy is indeed emergency resection but it is not always possible.
• ICP monitoring and DC are not commonly performed on brain tumor cases.
• The present case showed that ICP monitor and DC were helpful in times of ICU shortage.

## 1 Introduction

Meningioma comprises around 20–30 % of all central nervous system (CNS) tumors [1]. Despite the fact that meningioma is a slow-growing tumor [2], it can cause neurological emergency due to intracranial hypertension. Although emergency resection is the definitive treatment, it is not always practicable in some countries due to limited capacity and/or capability of emergency operating rooms [2].

Decompressive Craniectomy (DC) has been widely used worldwide for severe traumatic brain injury (TBI) [3] and stroke [4] to alleviate high intracranial pressure. In an international consensus regarding DC in TBI, most experts advocated the placement of intracranial pressure (ICP) monitor following DC [3]. The use of DC and ICP monitoring in cases of brain tumors is uncommon.

The availability of ICU is also among the deciding factors whether tumor resection is feasible to be performed [5]. ICU shortage during COVID-19 pandemic in several Asian countries including Indonesia has been reported [6]. In the present case, we report a meningioma patient in whom immediate meningioma resection was considered too risky due to ICU shortage during the COVID-19 pandemic and, therefore, underwent DC and ICP monitoring instead as life-saving measures. This procedure was conducted in the setting of academic hospital. This case report has been reported in line with the SCARE Criteria [7].

## 2 Case presentation

A 24-year-old man was brought in to the emergency room with a chief complaint of seizure. Prior to seizure, the patients vomited abruptly several times. The most recent seizure occurred 30 min prior to admission and lasted around 7 min. Since the seizure had ended, the patient had been rendered unconscious. Upon thorough history taking, it was revealed that the patient had no family with similar condition and was not on regular medications. Prior to this, the patient had complained of frequent headaches that seemed to be worsening and had gotten worse over the previous month. The patient has given his consent in written form for further examination.

**Fig. 1:**
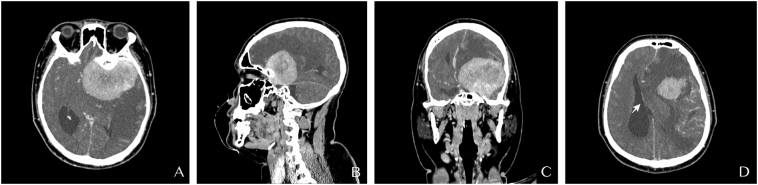
CT Scan with contrast showing extraaxial mass on the left sphenoid region, axial (A), sagittal (B), and coronal (C), which causes more than 2 cm of midline shift (arrow) and extensive perifocal edema (D).

Physical examination was notable for decreased consciousness (Glasgow Coma Scale (GCS) 11) and a dilated left pupil with intact light reflex. A contrasted Brain CT Scan revealed extra-axial mass on the left sphenoid with extensive tentacle edema, which pushed midline structures 2 cm toward the contralateral side (Fig. 1). The patient was then diagnosed with left sphenoid meningioma. No diagnostic challenge was found during examination in this case report.

Lowering the ICP was attempted using simple measures such as head elevation, corticosteroid, and mannitol. The patient's GCS deteriorated during observation and another seizure occurred. Emergency tumor resection was initially planned. However, the ICU confirmed that there were no available beds as the remaining ventilators were used for COVID-19 patients.

**Fig. 2:**
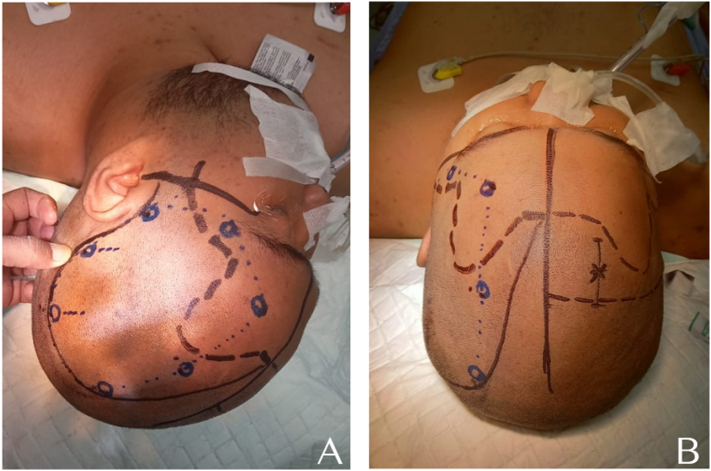
Design of the skin incision and the bone flap (A). The ICP monitor was inserted through the right Kocher point (B).

It was decided to perform intraparenchymal fiberoptic ICP monitor insertion and DC instead of tumor resection because tumor resection was considered too risky without the availability of ICU and given that patient's neurological status had deteriorated. The procedure was conducted immediately upon information of ICU by a senior trauma neurosurgeon in our institution. The ICP monitor was inserted through the Kocher point on the right side (Fig. 2). The initial pressure on the ICP monitor was 51 mmHg.

The DC was performed in a trauma case fashion: a question mark incision with a wide bone flap (Fig. 2) [3]. The ICP gradually decreased to around 35 mmHg after the bone flap was removed. Duramater was found to be tense. It was decided not to perform duraplasty because we thought it would cause the brain to bulge and lengthen the surgery time. After the surgery, we observed the ICP in the operating room before transferring the patient. The patient successfully regained spontaneous breathing and thus was extubated.

**Fig. 3:**
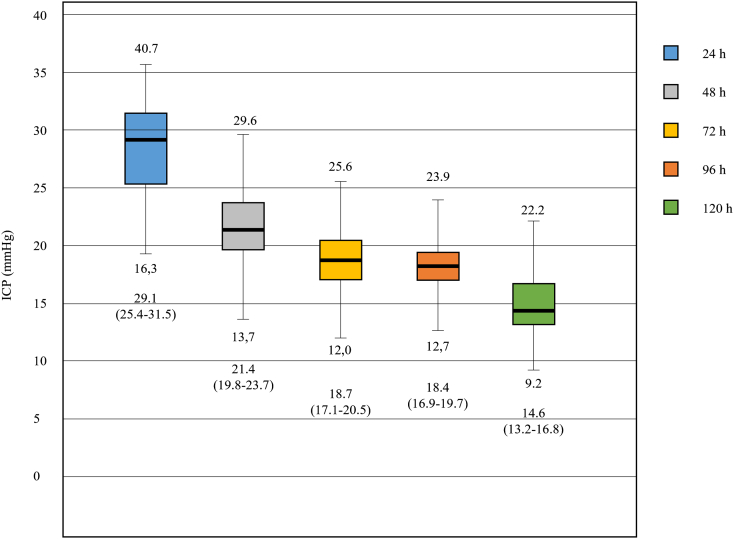
ICP data per 24-hour showed a decreasing trend. Data were extracted from the ICP monitor device.

The patient slowly regained consciousness in the recovery room after the procedure. The best observed GCS was 12. The skin overlying the decompressed site was bulged and the ICP remained around 30 mmHg. The patient's overall condition improved during our care and the ICP gradually returned to normal (Fig. 3) with the administration of ICP-directed mannitol and routine dexamethasone. Best observed GCS on fifth post-operative day was 15. The average ICP on the last post-operative day was 15.04 ± 2.7 mmHg. Two weeks afterwards, the patient came back to our outpatient clinic neurologically intact. The patient was then planned for elective tumor resection.

## 3 Discussion

In the case of a brain tumor, ICP monitoring and DC are not standard procedures. Both of these procedures are identic to traumatic brain injury (TBI) cases [3], [8]. The use of an ICP monitor has been linked to improved survivability in patients with severe TBI. To the best of our knowledge, reports on insertion of ICP monitor in brain tumor cases are non-existent. A recent study on ICP measurement in brain tumor cases utilized transcranial doppler instead. However, the authors of that study concluded that clinical signs of intracranial hypertension did not always imply an increase in ICP [9]. In contrast to this study, the present case showed compatibility of clinical features and actual ICP.

DC is recommended in TBI cases with intracranial hypertension, especially those with acute traumatic subdural hematoma [3]. This procedure is also rarely performed in brain tumor cases. In a study on six patients from Italy, DC was performed due to clinical and radiological worsening after meningioma resection [10]. Clavijo and colleague compiled reports on DC in various pathologies [11]. There were two studies which reported the use of DC in neuroblastoma [12] and brain angiometastasis from a non-seminomatous germ cell tumor [13].

The survival rate for meningioma patients after emergency DC craniectomy was 45 %, according to a case series involving 21 cases of meningiomas. These findings suggest that in patients with meningiomas who develop refractory postoperative edema, DC is an option worth considering as a salvage method, as it has the potential to save lives and has a good functional outcome in survivors [14]. However, there are also numerous known risks associated with DC. It has been reported that 53,9 % of patients experience complications. Poor neurological status and age are two patient-specific risk factors for developing complications (haemorrhages, postoperative infections, seizure). A low preoperative GCS (less than 8) has been linked to an increased risk of all types of complications. Another risk factor is being over the age of 65 [15]. Those literatures evidence demonstrates the advantages and disadvantages of the DC procedure in cases of central nervous system tumors.

DC was reported to decrease ICP from median values of 21.2 (18,7–24.2) to 15.7 (12.3–19.2) mmHg in a study [16]. Another study reported immediate plummeting of ICP from 35.0 (SD = 13.5) to 14.6 (SD = 8.7) mmHg after DC [16]. According to Brain Trauma Foundation. ICP levels above 22 mmHg should be considered critical [8], as these levels are associated with increased mortality and morbidity [17]. It's unclear whether this value applies to tumor cases as well. In the present case, the patient had ICP of 51 mmHg upon insertion of the fiberoptic intraparenchymal ICP monitor. After DC, the ICP dropped to 35 mmHg and dropped further over the next few days.

The decision to delay tumor resection was made due to ICU shortage. This unfortunate condition has been reported in various Asian countries including Indonesia during COVID-19 pandemic [6]. ICU reorganization during the pandemic mainly aimed to reduce capacity for elective surgery [18], but in fact adjustment might be made to accommodate unexpected COVID-19 care's needs. Given the circumstances, we chose surgical procedures that were shorter in duration and required less blood transfusions, allowing for non-ICU post-operative care. Surgery duration of more than four hours [19] is a predictor of ICU admission. Intraoperative blood loss and radiological evidence of mass effect and midline shift [5] prolonged ICU stay.

On fifth post-operative day, the patient was fully conscious with an average ICP of 15 ± 2.7 mmHg within the last 24 h. The patient was seen well at our outpatient clinic two weeks later and was prepared for elective tumor resection. The patient believes that each of decision the provider made will improve their quality of life. The doctor has been entrusted with the treatment that the patient requires. The patient and family are grateful for the success of the procedure performed at the time, and they continue to expect the best from our team.

## 4 Conclusion

ICP monitoring and DC are not commonly performed on brain tumor cases. However, in suboptimal situations, these procedures might save lives. The present case showed that ICP monitor and DC were helpful in times of ICU shortage.

## Provenance and peer review

Not commissioned, externally peer-reviewed.

## Funding sources

This article received no specific funding from any funding agency in the public, commercial, or non-profit sectors.

## Ethical approval

No ethical approval required.

## Consent

Written informed consent was obtained from the patient for publication of this case report and accompanying images. A copy of the written consent is available for review by the Editor-in-Chief of this journal on request.

## Research registration unique identifying number (UIN)

N/A.

## Guarantor

TA accepts full responsibility for this review manuscript.

## CRediT authorship contribution statement

Conception and design were done by TA, RM, EH, and HK. Supervision was done by TA. EH, HK, and RM collected the datas and materials. Analysis were done by EH and HK. Literature review and editing were done by RM and TA. All authors read and approved the final draft.

## Declaration of competing interest

The authors report there are no competing interests to declare.
